# Chronic Activation of AMPK Induces Mitochondrial Biogenesis through Differential Phosphorylation and Abundance of Mitochondrial Proteins in *Dictyostelium discoideum*

**DOI:** 10.3390/ijms222111675

**Published:** 2021-10-28

**Authors:** Malgorzata Heidorn-Czarna, Herbert-Michael Heidorn, Sanjanie Fernando, Oana Sanislav, Wieslawa Jarmuszkiewicz, Rupert Mutzel, Paul R. Fisher

**Affiliations:** 1 Department of Biology, Chemistry, Pharmacy, Institute for Biology-Microbiology, Freie Universität Berlin, 14195 Berlin, Germany; michael.heidorn@mac.com (H.-M.H.); rmutzel@zedat.fu-berlin.de (R.M.); 2 Department of Cellular Molecular Biology, Faculty of Biotechnology, University of Wroclaw, 50-383 Wroclaw, Poland; 3 Discipline of Microbiology, Department of Physiology, Anatomy and Microbiology, School of Life Sciences, College of Science, Health and Engineering, La Trobe University, Bundoora, VIC 3086, Australia; sgfernando@students.latrobe.edu.au (S.F.); O.Sanislav@latrobe.edu.au (O.S.); P.Fisher@latrobe.edu.au (P.R.F.); 4 Laboratory of Mitochondrial Biochemistry, Department of Bioenergetics, Faculty of Biology, Adam Mickiewicz University, 61-614 Poznan, Poland; wieslawa.jarmuszkiewicz@amu.edu.pl

**Keywords:** AMP-activated protein kinase, AMPK, mTORC1, mitochondrial biogenesis, mitochondrial phosphoproteome, phosphoproteomics, *Dictyostelium discoideum*

## Abstract

Mitochondrial biogenesis is a highly controlled process that depends on diverse signalling pathways responding to cellular and environmental signals. AMP-activated protein kinase (AMPK) is a critical metabolic enzyme that acts at a central control point in cellular energy homeostasis. Numerous studies have revealed the crucial roles of AMPK in the regulation of mitochondrial biogenesis; however, molecular mechanisms underlying this process are still largely unknown. Previously, we have shown that, in cellular slime mould *Dictyostelium discoideum*, the overexpression of the catalytic α subunit of AMPK led to enhanced mitochondrial biogenesis, which was accompanied by reduced cell growth and aberrant development. Here, we applied mass spectrometry-based proteomics of *Dictyostelium* mitochondria to determine the impact of chronically active AMPKα on the phosphorylation state and abundance of mitochondrial proteins and to identify potential protein targets leading to the biogenesis of mitochondria. Our results demonstrate that enhanced mitochondrial biogenesis is associated with variations in the phosphorylation levels and abundance of proteins related to energy metabolism, protein synthesis, transport, inner membrane biogenesis, and cellular signalling. The observed changes are accompanied by elevated mitochondrial respiratory activity in the AMPK overexpression strain. Our work is the first study reporting on the global phosphoproteome profiling of *D. discoideum* mitochondria and its changes as a response to constitutively active AMPK. We also propose an interplay between the AMPK and mTORC1 signalling pathways in controlling the cellular growth and biogenesis of mitochondria in *Dictyostelium* as a model organism.

## 1. Introduction

Mitochondria are involved in a vast array of biological processes, including energy transformation, calcium signalling, iron–sulphur (Fe-S) cluster biosynthesis, redox balance, the formation of reactive oxygen species (ROS) and the regulation of the apoptotic program, which are fundamental for cell metabolism and survival. The maintenance of mitochondrial homeostasis depends on the continuous fusion and fission of the mitochondrial network and a proper balance between mitochondrial biogenesis and degradation via mitophagy. The highly flexible nature of mitochondria allows the organelles to respond to cellular needs and environmental signals by changing their morphology, mass, content and dynamics.

Mitochondrial biogenesis is a complex and largely controlled process that occurs through the growth and division of existing mitochondria. It requires the increased and coordinated expression of nuclear and mitochondrial genomes, as well as synchronized import and the assembly of proteins and phospholipids in the mitochondrial membranes. The regulation of mitochondrial biogenesis occurs at transcriptional, translational and post-translational levels [1]. It depends on diverse signalling pathways which respond to hormonal, environmental or developmental signals and changes in the energy status of the cell [2]. Long-term exercise training, which is associated with energy deprivation and chronic metabolic stress, promotes mitochondrial biogenesis and increases muscle oxidative capacity to generate ATP [3,4,5]. Zong et al. [6] demonstrated that the reduction in the cellular ATP/AMP ratio resulted in the activation of AMP-activated protein kinase (AMPK) and increased mitochondrial biogenesis in mice skeletal muscles. The authors further reported that AMPK activation led to an increased expression of peroxisome proliferator-activated receptor γ coactivator 1α(PGC-1α) and calcium/calmodulin-dependent protein kinase IV (CaMKIV), both recognized as major regulators of mitochondrial biogenesis [7,8]. 

Phosphorylation is the most common reversible post-translational modification of proteins that alter their structural conformation and function, providing cells with a mechanism of response to chemical signals. AMPK is a serine/threonine protein kinase present in virtually all eukaryotic organisms [9,10,11,12,13,14]. It is a critical metabolic stress-responsive enzyme that acts at a central control point in cellular energy homeostasis. Upon low energy conditions, AMPK is activated and redirects metabolism by switching off the ATP-consuming anabolic pathways and switching on ATP-producing catabolic pathways through the reversible phosphorylation of multiple proteins to restore energy homeostasis [15,16]. Numerous studies have revealed the diverse roles of AMPK in regulating various cellular functions, such as cell growth and proliferation, metabolism, cell polarity and migration, and autophagy [17]. AMPK is also implicated in the regulation of mitochondrial homeostasis: mitochondrial biogenesis and mitophagy, as well as mitochondrial fission [18,19]. The phosphorylation of at least two known mitochondrial outer membrane proteins by AMPK (MFF—mitochondrial fission factor, and ACC2—acetyl-CoA carboxylase 2) [19,20,21] and the impact of AMPK on mitochondrial dynamics suggest that AMP-activated kinase colocalizes temporarily with the mitochondria. Indeed, it has been shown that, in humans, N-myristoylation of the AMPKβ subunit is essential for AMPK association with mitochondria and the removal of damaged mitochondria by mitophagy [22]. Interestingly, Miyamoto et al. [23] revealed the existence of AMPK at the mitochondria, independently from its cytosolic localization, using organelle-specific small-peptide probes for AMPK activity. 

Research on the role of AMPK on mitochondrial homeostasis has so far focused mainly on mammalian cells and AMPK as a potential target for treating metabolic and cardiovascular diseases and cancer. In this work, we used the cellular slime mould *Dictyostelium discoideum*, an established eukaryotic model for studying mitochondrial biology and disease [11,24,25,26], to provide insight into how AMPK regulates the biogenesis of mitochondria. Previous research documented that a *D. discoideum* strain, overexpressing the catalytic AMPKα subunit, suffered morphological and developmental alterations, which include decreased growth rate both on agar plates with bacterial food sources and in liquid medium, decreased phototaxis and thermotaxis, decreased stalk length and increased stalk width, as compared with the wild-type, Ax2 cells [11,27]. These phenotypic abnormalities, seen in the AMPKα-overexpressing cells, were accompanied by enhanced mitochondrial biogenesis and increased intracellular levels of ATP [11]. 

**Figure 1:**
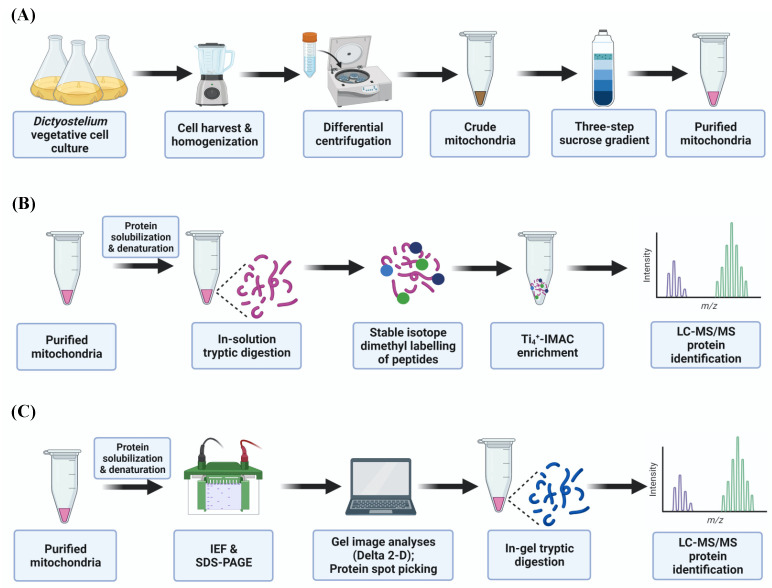
Experimental workflow used in this study. (**A**) Vegetatively growing *Dictyostelium discoideum* wild-type (WT) and AMPKα-overexpressing (HPF444) cells were harvested at the exponential phase of growth and subjected to isolation and purification of mitochondria by differential centrifugation and sucrose density gradient centrifugation, respectively; (**B**) purified mitochondria were subjected to solubilization and tryptic digestion. Phosphopeptides were enriched via immobilized titanium (IV) ion affinity chromatography (Ti_4_^+^-IMAC) and identified by LC-MS/MS; (**C**) purified mitochondrial fractions were solubilized and resolved by 2D-PAGE (IEF/SDS-PAGE) electrophoresis. Proteins displaying a significant difference in abundance were picked from the gels, designed for in-gel tryptic digestion and peptide identification via MS/MS. More details on the survey design of the experiment are given in the Material and Methods as well as Results sections. Created with BioRender.com.

The purpose of this study was to determine the impact of chronically activated AMPKα on the phosphorylation state and the abundance of mitochondrial proteins in *D. discoideum* cells, and thus to identify potential downstream protein targets that lead to increased mitochondrial biogenesis (Figure 1). By applying mass spectrometry-based phosphoproteomics of *D. discoideum* mitochondria, we identified 103 phosphoproteins and, among them, 35 proteins displayed variations in phosphorylation caused by chronic AMPKα activity. Differential phosphorylation levels showed proteins associated with oxidative phosphorylation (OXPHOS), transport, protein synthesis, and cellular signalling. Our 2-D PAGE proteomic analyses highlighted that, in *D. discoideum*, the enhanced mitochondrial biogenesis caused by the chronic activity of AMPKα is associated with an increased abundance of proteins related to protein translation and folding, amino acid degradation, mitochondrial inner membrane biogenesis and mtDNA metabolism. Our work is the first study reporting on the global phosphoproteome profiling of *D. discoideum* mitochondria and its changes as a response to enhanced mitochondrial biogenesis caused by constitutively active AMPK.

## 2. Results

### 2.1. Identification of Phosphorylation Sites in Dictyostelium Mitochondrial Proteins

**Figure 2:**
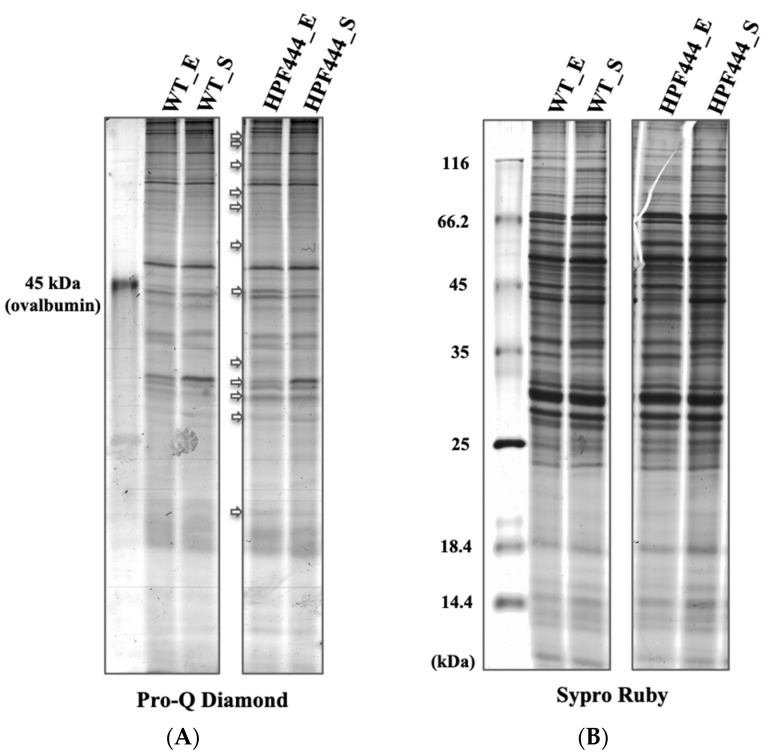
Phosphoprotein (Pro-Q Diamond) and total protein (Sypro-Ruby) 1-D SDS-PAGE gel stain of total mitochondrial proteins isolated from vegetative cells of *D. discoideum* WT and HPF444 Scheme. (**A**) A total of 100 µg of purified mitochondrial proteins isolated from vegetative cells from the exponential (E) and stationary (S) phases of growth (WT_E, WT_S; HPF444_E, HPF444_S) were separated by 12% SDS-PAGE and stained with Pro-Q Diamond (Thermo Fisher Scientific, Waltham, MA, USA) to selectively visualize phosphorylated proteins; (**B**) The gel stained with Pro-Q Diamond was further stained with Sypro Ruby (Thermo Fisher Scientific, Waltham, MA, USA) to detect total mitochondrial proteins. The Unstained Protein Molecular Weight Marker (Thermo Fisher Scientific, Waltham, MA, USA) was used. The arrows indicate the phosphoproteins that show different intensity in the mutant in comparison to the WT mitochondria.

To determine whether there is an impact of chronic AMPKα activation on the phosphorylation level of mitochondrial proteins in *Dictyostelium* cells, we first separated total mitochondrial proteins obtained from vegetative WT and AMPKα-overexpressing cells (HPF444) harvested at the exponential (E) and stationary (S) phases of growth by polyacrylamide gel electrophoresis in SDS (SDS-PAGE) (Figure 2). To selectively detect mitochondrial phosphoproteins, we stained the gel with Pro-Q Diamond dye, which discriminates between phosphorylated and non-phosphorylated proteins in acrylamide gels [28,29]. As demonstrated in Figure 2A, the phosphoproteins were explicitly detected in both WT and HPF444 mitochondria; however, a few protein bands showed either higher or lower intensity in the mutant compared to the WT, especially in the mitochondria of exponentially growing cells. Subsequent staining of the gels with Sypro Ruby dye excluded differences in the total protein of WT and HPF444 samples separated on that gel (Figure 2B). These data indicated the presence of differential phosphorylation of mitochondrial proteins in *Dictyostelium* AMPKα-overexpressing cells and prompted the performance of a large-scale quantitative comparative proteomic investigation of mitochondrial phosphoproteomes.

**Figure 3:**
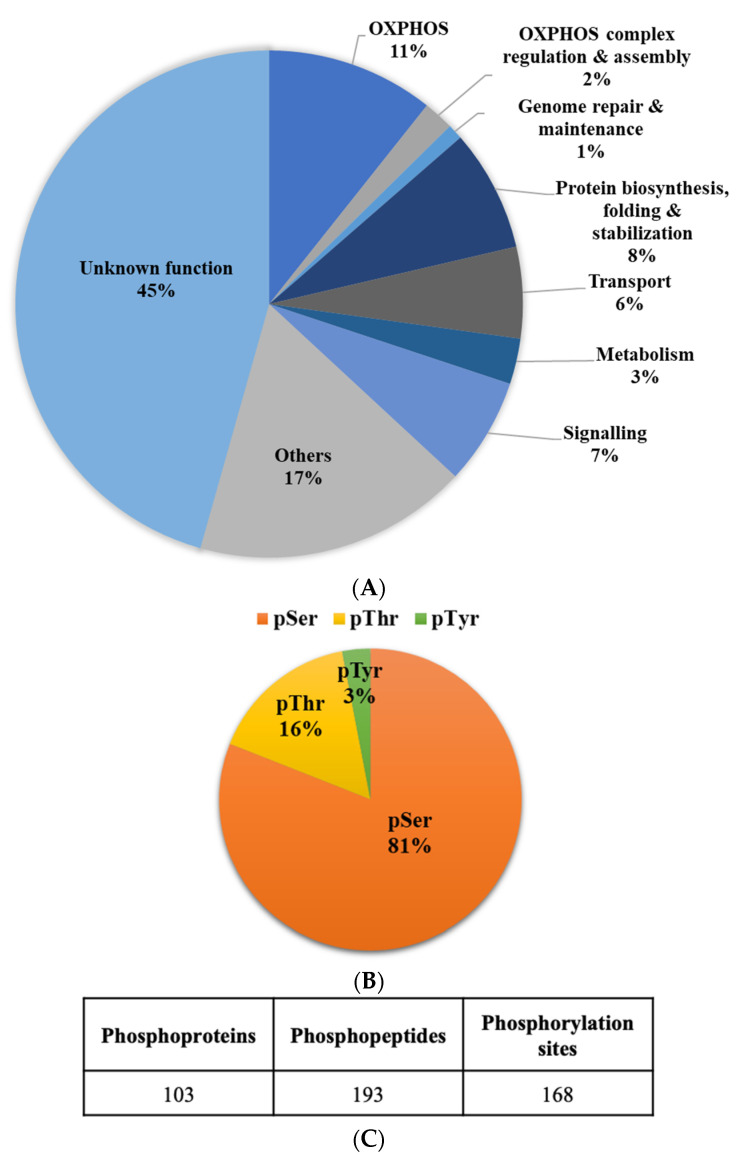
Profiling phosphoproteins of *D. discoideum* mitochondria. (**A**) Proportion and functional categories of identified phosphoproteins in mitochondrial preparations; (**B**) distribution of serine (pSer), threonine (pThr), and tyrosine (pTyr) phosphorylation sites in identified proteins; (**C**) the number of non-redundant phosphoproteins, phosphopeptides and phosphorylation sites identified in mitochondrial preparations from *D. discoideum* WT and HPF444 strains.

Consistent with our previous studies [11], we used exponentially growing cells of *D. discoideum* WT and HPF444 strains for mitochondrial phosphoproteome analyses. The phosphorylated proteins of purified mitochondrial fractions were investigated using titanium (IV) ion affinity chromatography (Ti_4_^+^-IMAC) for the selective enrichment of phosphopeptides in combination with an LC-MS/MS workflow for protein identification. Overall, our mitochondrial phosphoproteome analyses led to the identification of a total of 193 unique phosphopeptides, corresponding to 168 phosphorylation sites in 103 mitochondrial proteins in both WT and mutant cells (Figure 3, Table 1 and Table S1). We found 136 phosphoserine residues, 27 phosphothreonine residues, and only 5 phosphotyrosines among a total of 168 phosphorylation sites, which gives 81% pSer, 16% pThr and 3% pTyr (Figure 3B, Table 1 and Table S1). The identified phosphoproteins were categorized into the following nine functional groups: (1) OXPHOS, (2) OXPHOS complex regulation and assembly, (3) Genome repair and maintenance, (4) Protein synthesis, folding and stabilization, (5) Transport, (6) Signalling, (7) Metabolism, (8) Other functions, and (9) Unknown functions (Figure 3A, Table 1 and Table S1). The largest group of phosphoproteins (n = 47) was identified among the proteins with unknown functions (Table S1). These uncharacterized proteins were designated as proteins of unknown function because no detectable homology to proteins of known functions at the sequence and structure level was found to date. Notably, numerous phosphorylation sites (*n* = 20) were also identified among the OXPHOS I-V complexes, especially in the protein subunits of cytochrome *b-c*1 complex (complex III), cytochrome *c* oxidase (complex IV) and ATP synthase (complex V). The ATP synthase subunits 4 and beta were phosphorylated at multiple sites (n = 3 and n = 6, respectively), indicating that, in *D. discoideum* mitochondria, the ATP synthase complex is a target for phosphorylation-mediated regulation of its activity and/or structure. Interestingly, among the phosphorylated proteins functionally related to OXPHOS, we found F1F0-ATPase putative regulatory protein IF1 phosphorylated in serine 81 (Ser-81). This protein probably functions as the physiological inhibitor of the mitochondrial ATP synthase. In humans, the phosphorylation of IF1 in serine 39 (Ser-39) by mitochondrial protein kinase A (PKA) prevents its binding to the ATP synthase [30]. The identified phosphosite in *Dictyostelium* IF1 does not match the phosphorylated Ser residue in the human orthologue (Table S2).

Another important phosphorylated protein group was that of those involved in mitochondrial transport (n = 6) (Table 1). In this group, the most striking was mitochondrial substrate carrier family protein adenine nucleotide translocase (ANT), an orthologue of the human ADP/ATP translocase 1 (SLC25A4), with several phosphorylated serines (Ser-16, Ser-44, Ser-46, Ser-152, Ser-308) and threonines (Thr-47, Thr-150) identified. In humans, the ADP/ATP translocase 1 has also been phosphorylated at multiple sites. However, among phosphorylated amino acids identified in the *D. discoideum* ANT, only one (Ser-46) corresponds to the often-phosphorylated serine of the human orthologue (Ser-42) (Table S2) [PhosphoSitePlus, phosphosite.org]. Leucine zipper-EF-hand-containing transmembrane protein 1 (LETM1) and EF-hand domain-containing protein, which, similar to human LETM1, has been designated as a protein involved in the maintenance of mitochondrial osmotic balance, morphology, and viability [reviewed in 31], were found to be phosphorylated in threonine 282 (Thr-282) in *D. discoideum* mitochondria (Table 1). In mitochondria, LETM1 functions directly as a K^+^/H^+^ exchanger and indirectly modulates Ca^2+^ flux by affecting the Ca^2+^ cycle [31]. Recently, the phosphorylation of human LETM1 by PTEN-induced kinase 1 (PINK1) at Thr-192, which corresponds to Thr-178 in *D. discoideum* (Table S2), has been reported to facilitate calcium transport in neuronal mitochondria [32]. 

In the present study, we identified several phosphopeptides of a protein classified into mitochondrial metabolism, i.e., CDGSH iron–sulfur domain-containing protein, an orthologue of human mitoNEET-related protein 2 (Table 1). mitoNEET is a mitochondrial outer membrane protein that may be involved in the biogenesis of Fe-S clusters and electron transfer from FMNH2 to oxygen or coenzyme Q [33]. Notably, in *Dictyostelium*, this protein was found to be phosphorylated at serine 75 (Ser-75), which corresponds to the frequently phosphorylated Ser-83 in the human mitoNEET-related protein 2 (Table S2) [34,35,36]. Interestingly, in the mitochondria of *D. discoideum*, we found two sites for the phosphorylation of carbonic anhydrase, a kinase-regulated enzyme involved in ammonia detoxification and glucose metabolism in mammalian mitochondria [37,38].

Among the phosphoproteins we identified, a number are associated with various signalling pathways (Table 1), several of them carrying phosphorylated tyrosines, including glycogen synthase kinase-3 (GSK3), dual-specificity protein kinase SHKA, dual-specificity protein kinase SHKD, and a signal transducer and activator of transcription A (STATa). Phosphotyrosines constitute only a small proportion among the total number of phosphorylated amino acids identified in this work (5 pTyr in comparison to 27 pThr and 136 pSer) (Figure 3B). Here, we have shown that STATa is phosphorylated at the tyrosine residue at position 702 (Tyr-702) (Table 1), in agreement with previous studies by Kawata et al. [39]. In the group of proteins classified into the signalling category, we also included a protein similar to human Ragulator complex protein LAMTOR1; however, its direct mitochondrial colocalization has not been shown so far. We found that in *Dictyostelium*, this protein was phosphorylated at multiple sites (Thr-34, Ser-36, Ser-56, Ser-172). Among them, the Ser-36 residue corresponds to phosphorylated threonine (Thr-30) of the human orthologue (Supplemental Table S2) [36]. 

### 2.2. Protein Kinases Associated with Dictyostelium Mitochondria

Of all the identified mitochondrial phosphoproteins, the presence of several protein kinases (GSK3; Severin kinase, SvkA; SHKA and SHKD) in *D. discoideum* mitochondrial fractions is of great interest (Table 1 and Table 2). These kinases represent known kinase groups, i.e., CMGC (named after serine/threonine CDK, MAPK, GSK3, and CLK protein kinase families), STE (that includes serine/threonine Mitogen-Activated Protein Kinase families STE7, STE11, and STE20), and TKL (Tyrosine Kinase-Like protein kinase families) (Table 2) [40]. To date, none of these enzymes have been reported to be localized in *Dictyostelium* mitochondria; however, due to their similarity to the mammalian protein kinase groups, it is possible that at least a fraction of each of these enzymes resides in or on the slime mould mitochondria, or is functionally associated with them [41]. 

GSK3, which is an orthologue of the human GSK-3B kinase, has been detected as phosphorylated at serine 213 (Ser-213) and tyrosine 214 (Tyr-214) (Table 1). These phosphorylated amino acids correspond to the ubiquitously phosphorylated Ser-215 and Tyr-216 in human GSK-3B (Table S2). The same phosphorylated sites have been previously detected by Nichols et al. [42] in a global phosphoproteomic analysis of *Dictyostelium* vegetative cells responding to different chemoattractants, while the Ser-213 phosphorylation site was found in the study of Sugden et al. [43] as the response of *Dictyostelium* cells to differentiation-inducing factor-1 (DIF-1). Previously, it has been shown that in *Dictyostelium*, Tyr-214 and Tyr-220 of the GSK3 kinase are targets for cAMP-regulated phosphorylation and both sites are critical for GSK3 activation [44]. 

Additionally, we uncovered the phosphorylation of the SHKA kinase at the tyrosine residue 525 (Tyr-525), which corresponds to the Tyr-533 of its human orthologue TAK1 (MAP3K7) kinase, and the phosphorylation of SHKD at the tyrosine residue 739 (Tyr-739) (Table 1 and Table S2). To date, there is no information on the phosphorylation of these sites in *D. discoideum* or humans to date. Another identified kinase, Severin kinase (SvkA), which is also known as Hippo related kinase-svk (Krk-svk) [45], was phosphorylated at serine 375 (Ser-375) (Table 1). Previously, Ca^2+^-dependent autophosphorylation of SvkA in axenically growing *Dictyostelium* cells was demonstrated by Eichinger et al. [46]. This same phosphosite (Ser-375) in SvkA was also detected in *Dictyostelium* responding to cAMP by Nichols et al. [42].

### 2.3. Differential Phosphorylation Responses Caused by the Constitutive Hyperactivity of AMPKα

It has been known for some time that active AMPK promotes mitochondrial biogenesis [47]. In this work, the use of an MS-based quantitative phosphoproteomic approach has enabled us to detect differentially phosphorylated mitochondrial and mitochondria-associated proteins in response to chronically active AMPKα (Table 3 and Table S3). We focused on these phosphorylated proteins, which were identified in at least two biological replicates or with differential phosphosites identified in at least two peptides. These proteins are presumably downstream targets of the AMPK activity in *Dictyostelium* cells. Of all the identified differing phosphoproteins that were reproducibly quantified, only four proteins showed higher phosphorylation levels, and 31 had decreased phosphorylation levels in the HPF444 mitochondria, compared to WT mitochondria (Table 3 and Table S3). These differentially phosphorylated proteins were unexpectedly over-represented by uncharacterized proteins (2 with higher and 12 with lower phosphorylation level), making it difficult to fully interpret phosphorylation responses to disturbed AMPK signalling.

One of the proteins with a higher phosphorylation level in the mutant mitochondria is LETM1 (Leucine zipper-EF-hand-containing transmembrane protein 1), found phosphorylated at the Thr-282 residue (Table 3). Numerous findings using yeast, *Drosophila*, and mammalian mitochondria indicate the importance of LETM1 in mitochondrial K^+^ and Ca^2+^ homeostasis, and, thus, osmotic balance, affecting mitochondrial morphology, dynamics, and metabolism [48,49,50,51,52,53]. Additionally, it has also been suggested that, in yeast and mammals, LETM1 is a ribosomal-binding protein and has a function in mitochondrial translation, assembly of respiratory chain complexes, and respiration [54,55,56]. Whether LETM1 has a critical function in *Dictyostelium* mitochondria is currently unknown.

Another differentially modified protein, S5 DRBM domain-containing protein, had an increased phosphorylation level at serine 1587 (Ser-1587). In contrast, other identified phosphosites did not show differences in phosphorylation compared to the WT mitochondria (Table 1 and Table 3). The presence of the S5 DRBM domain and the similarity to other organisms indicate that this protein is likely *Dictyostelium* mitochondrial ribosomal protein S5, a component of a small 28S subunit. However, this must be confirmed. Nonetheless, these results indicate that the mitochondrial protein translation machinery components are most likely affected by chronically active AMPK to stimulate mitochondrial biogenesis.

Our data showed that, aside from a large number of uncharacterized proteins (n = 12), a decrease in steady-state phosphorylation level in response to the chronic activity of AMPK was also observed for the OXPHOS components (cytochrome *c*1, Ser-112; ATP synthase subunit gamma, Thr-85; ATP synthase subunit beta, Thr-69), chaperones and cochaperones (heat shock protein 70 kDa, Thr-655; DnaJ family protein, Ser-213), proteins involved in mitochondrial transport (mitochondrial substrate carrier family protein ANT, Ser-152, 308; mitochondrial substrate carrier family protein X, Ser-87) as well as an orthologue of human CISD3 (mitoNEET-related protein 2, Ser-75) (Table 3). All of these differentially phosphorylated mitochondrial proteins are either located in the inner membrane or matrix, except for mitoNEET-related protein 2, which by similarity to its human orthologue is likely located in the outer membrane of *D. discoideum* mitochondria [57].

Notably, among proteins with decreased phosphorylation levels, we also found non-mitochondrial proteins categorized in this study in the intracellular signalling category, such as SHKA protein kinase (Tyr-525), a protein similar to the human Ragulator complex protein LAMTOR1 (Thr-34, Ser-56, 172) and Ras-related protein Rab-1A (Ser-76) (Table 1 and Table 3). These proteins might constitute either upstream or downstream players of the AMPK pathways, regulating mitochondrial biogenesis and activity.

### 2.4. Effect of Chronically Elevated AMPKα Activity on the Abundance of the Mitochondrial Proteins

**Figure 4:**
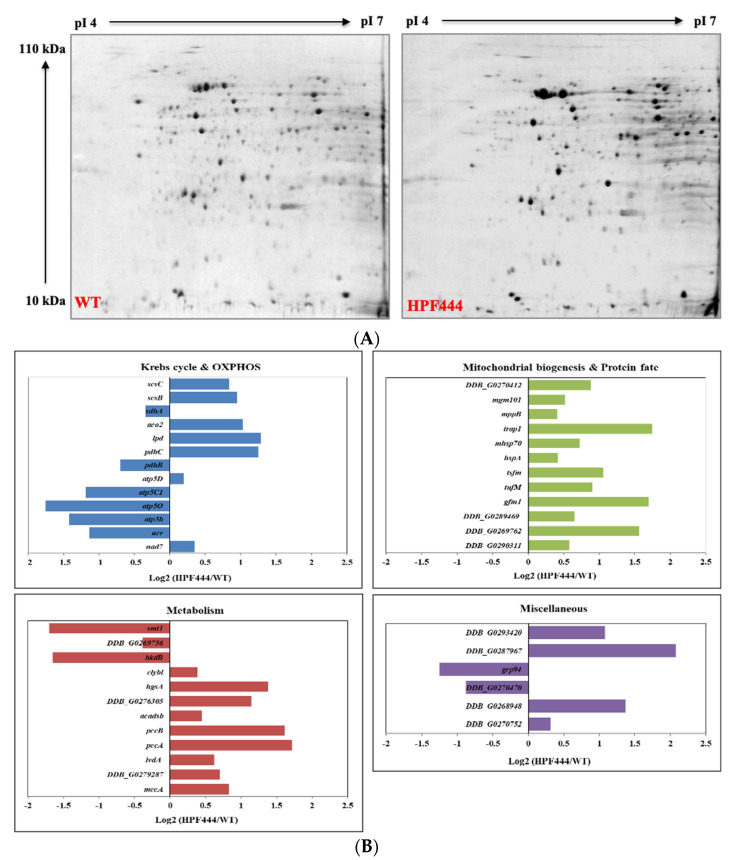
(**A**) Representative Coomassie brilliant blue (CBB)-stained 2-D PAGE gels of total mitochondrial proteins isolated from exponentially growing vegetative cells of *D. discoideum* WT and HPF444 strains. A total of 400 µg of purified mitochondrial proteins was separated by 2-D PAGE and stained by colloidal CBB. Three independent biological replicates of each strain were analysed using Delta 2-D Software 4.2 (DECODON, Greifswald, Germany); (**B**) Relative levels of mitochondrial proteins identified by 2-D PAGE analysis in *D. discoideum* HPF444 strain compared to WT cells. The relative abundance of proteins is expressed as Log2 ratios. In italics, gene name of a differing protein is presented. Full names of proteins and detailed statistical analysis are given in Table S4.

We also determined the effect of constitutive hyperactivity of the AMPKα subunit on the total mitochondrial proteome by identifying differentially expressed proteins using two-dimensional polyacrylamide gel electrophoresis (2-D PAGE). For the quantitative studies, mitochondrial proteins isolated from exponentially growing WT and HPF444 vegetative cells were analysed in three independent experiments (Figure 4A). Protein spots that showed a significant difference in abundance between HPF444 vs. WT (fold difference of ±1.2, *p* ≤ 0.05) were manually excised from Coomassie-stained master gels and identified by mass spectrometry. The identities of the differing proteins are presented in Table S4. Various energy-conserving proteins (the pyruvate dehydrogenase complex (PDH), the Krebs cycle and OXPHOS components), mitochondrial membrane biogenesis proteins and genome maintenance proteins, enzymes involved in protein synthesis, folding, and stabilization, diverse enzymes for amino acid and lipid metabolism, and some hypothetical proteins responded to the chronic activity of AMPK. Notably, our results show the remarkable plasticity of *D. discoideum* mitochondrial energy metabolism, as most of the identified enzymes of the PDH and the Krebs cycle, as well as enzymes involved in the degradation of amino acids and fatty acids, were significantly upregulated. On the other hand, the identified subunits of cytochrome b-c1 complex (Rieske Fe-S protein) and ATP synthase (ATP synthase subunits beta, OSCP and gamma) showed a strong down-regulation (Figure 4B). However, the most striking changes in abundance were observed among proteins involved in protein synthesis and folding. All of the identified mitochondrial ribosomal proteins (L17, S18 and S15P), mitochondrial translation elongation factors (G, Tu and Ts), as well as mitochondrial chaperones (chaperonin 60, Hsp70 and Hsp90 family protein TRAP1) were largely upregulated in the HPF444 mutant mitochondria (Figure 4B). These results indicate that enhanced mitochondrial protein synthesis and folding caused by overexpression of a constitutively active AMPK are among the critical signs of increased mitochondrial biogenesis. This assumption is also supported by an almost two-fold higher amount of mitofilin (Mic60), a protein essential for the mitochondrial inner membrane organization determining mitochondrial morphology and mitochondrial DNA (mtDNA) organization in mammals [58], as well as a 1.4-fold increase in the mitochondrial genome maintenance protein 101, which, in *Saccharomyces cerevisiae*, has been shown to bind directly to mtDNA and be essential for its maintenance and repair (Figure 4B, Table S4) [59].

Among these differentially expressed proteins, there were only four polypeptides which were also characterized by differential phosphorylation levels in the HPF444 mitochondria (Table 3). The Hsp70 protein was more abundant but showed lowered phosphorylation levels in HPF444. The hypothetical protein DDB0306113 displayed decreased abundance, but its phosphorylation level was significantly higher. For the ATP synthase gamma and beta subunits, which were less abundant in the mutant mitochondria, the level of phosphorylation varied in the same direction as protein amounts (Table 3, Tables S3 and S4).

Overall, these results indicate that, in *Dictyostelium* cells, the enhanced mitochondrial biogenesis due to chronic activity of AMPKα is associated with an increased abundance of proteins related to amino acid degradation, protein translation and folding, mitochondrial inner membrane biogenesis and mtDNA metabolism, as well as the differential phosphorylation of selected mitochondrial and mitochondria-related proteins, especially those involved in energy conservation, protein synthesis and cellular signalling.

### 2.5. Effect of Chronically Elevated AMPKα Activity on Mitochondrial Respiratory Function

**Figure 5:**
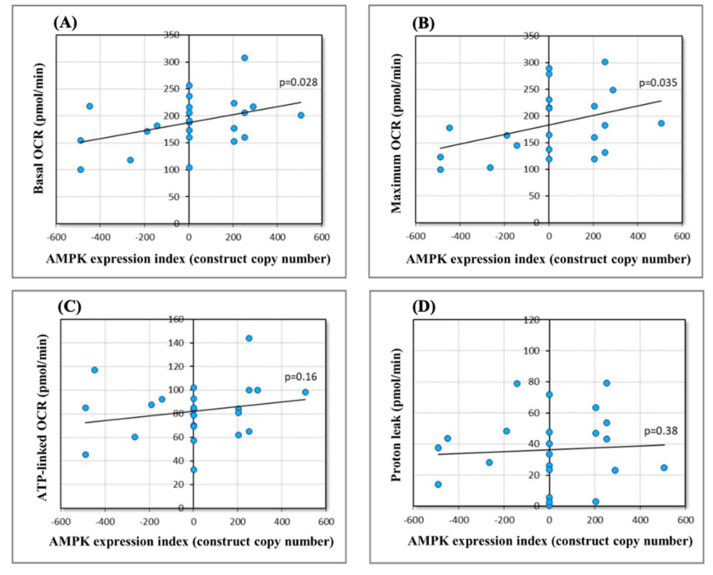

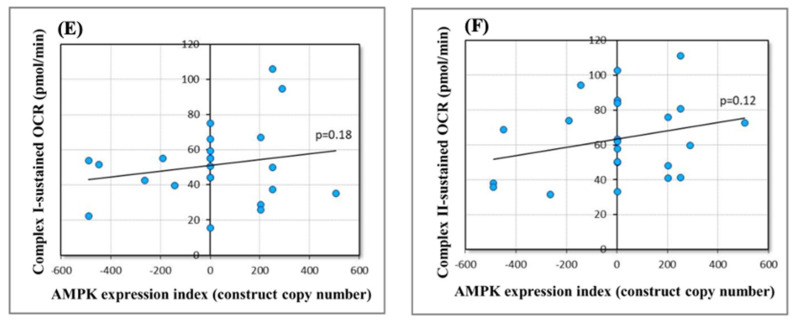
Effect of AMPK expression levels on mitochondrial respiratory function. Respiratory O_2_ consumption rates (pmol/min) were assayed using 1 × 10^5^ amoebae per well of the wild-type parental strain (A × 2) or strains stably transformed with either an AMPK antisense-inhibition or overexpression construct. A predetermined convention was followed using the copy number of the construct as an expression index (negative numbers for antisense inhibition and positive numbers for overexpression). To estimate the contribution of the ATP-linked oxygen consumption rate (OCR) (**C**) and non-ATP-linked OCR (proton leak) (**D**) to the basal respiratory rate (**A**), N,N′-dicyclohexylcarbodimide (DCCD) was added to inhibit ATP synthesis at Complex V. Subsequently, a protonophore (uncoupler) carbonyl cyanide 3-chlorophenol hydrazone (CCCP) was added to determine the maximum OCR (**B**). The subsequent addition of rotenone (Complex I inhibitor) and either antimycin A (Complex III inhibitor) or benzohydroxamic acid (BHAM, AOX inhibitor) allowed to estimate the contribution of Complex I (**E**) and Complex II (Complex II/III + Complex II/AOX) (**F**) to the maximum OCR. The slope of the regression line was positive as assumed in all cases, reaching statistical significance in the case of the basal OCR and the maximum uncoupled OCR.

After demonstrating that chronically elevated AMPK activity induces changes in the abundance and phosphorylation state of mitochondrial proteins in *Dictyostelium*, we tested the functional implications of these changes for mitochondrial respiration. Mitochondrial respiratory activity is regulated homeostatically by AMPK as part of an extended network of signalling pathways. This regulation is exerted at the level of transcription and the translation of mitochondrial proteins, the activity of key proteins involved, and the supply of oxidizable substrates. We hypothesized that chronically elevated AMPK activity would result in a new steady state, with elevated mitochondrial respiratory activity. To test this, we measured mitochondrial respiratory function using Seahorse respirometry, as described previously [60], using strains with different copy numbers of AMPK antisense-inhibition or overexpression constructs (Table S5) [11]. Both basal respiration rates (Figure 5A) and the maximum respiratory capacity of the electron transport chain (Figure 5B) increased significantly with increasing levels of AMPK alpha subunit expression. These results confirm that the observed changes to the mitochondrial phosphoproteome in AMPK overexpression strains are accompanied by elevated mitochondrial respiratory activity. The individual components of basal mitochondrial respiration (ATP synthesis and proton leak, Figure 5C,D) and maximum mitochondrial respiratory capacity (activity of Complexes I and II, Figure 5E,F) showed the same trends, but did not reach statistical significance (Table S5). This is likely because the sample sizes (number of strains) did not provide sufficient statistical power, given the necessarily smaller magnitude of each individual component and its correspondingly smaller effect on measured respiration rates.

## 3. Discussion

We performed the first profiling of the mitochondrial phosphoproteome in *D. discoideum* vegetative cells and its alterations in response to the chronic activation of the AMPK catalytic subunit. Using titanium ion affinity chromatography for selective phosphopeptide enrichment, in combination with high-performance LC-MS/MS, we identified 168 phosphorylation sites in 103 proteins of isolated mitochondria of both wild-type and mutant cells. The distribution between phosphorylated residues of the mitochondrial proteins was 81% pSer, 16% pThr and 3% pTyr, which is similar to the mitochondrial proteomes of mammalian cells, independent of the tissue or cell used for mitochondria isolation (82% pSer, 15% pThr, and 3% pTyr) [41], and less compared to mouse mitochondria (90% pSer, 9% pThr, 1% pTyr) [61]. All identified phosphorylated mitochondrial proteins are nuclear-encoded, suggesting that, in *Dictyostelium*, the phosphorylation of mitochondrial DNA-encoded proteins is rare.

There are a variety of functional consequences of phosphorylation and dephosphorylation of mitochondrial proteins, including their location, proper metabolism, the functionality of the Krebs cycle and OXPHOS, signalling, processes of fission and fusion, as well as the decision for mitophagy and apoptosis, reviewed in [62,63]. In this work, a substantial number of phosphorylation sites (besides the largest group of identified phosphosites among proteins with unknown function) was found within the OXPHOS components, particularly complexes III (subunits 6 and 8, heme and Rieske proteins), IV (subunits 4 and 5) and V (subunits 4, gamma, OSCP and beta) (Table 1). Despite a large amount of data showing the phosphorylation of most cytochrome *b-c*1 subunits, little is known about the functional significance of these modifications [64]. One of the two identified phosphosites in the cytochrome *c*1 (heme protein) subunit, Ser-112, was shown to be down-regulated in the *Dictyostelium* AMPKα-overexpressing mitochondria (Table 3).

There are multiple lines of evidence revealing that, in various organisms, the phosphorylation of the ATP synthase components impacts the activity and/or stability of this complex. In humans, several phosphosites of the ATP synthase beta subunit have been identified [65,66,67], and phosphorylation of some specific sites appears to be crucial for complex dimer assembly and activity. Kane et al. [68] used *S. cerevisiae* as a model system to understand the functional consequences of beta subunit phosphorylation in cardiac mitochondria. They showed that mutations of Thr-262 resulted in the down-modulation of the ATP synthase activity, while mutations of the Thr-58 impaired dimer formation, leading to decreased activity and impairing the crucial role of ATP synthase in cristae formation and overall mitochondrial shape [69]. Among the multiple phosphosites detected in the *Dictyostelium* ATP synthase beta subunit (Table 1), the phosphorylation of the Thr-69 residue was reduced in the HPF444 mutant (Table 3). Based on the comparison of the position of Thr-69 in the *D. discoideum* beta subunit to that of *S. cerevisiae* and human enzymes, it is highly probable that, in slime mould, the Thr-69 phosphosite is also located on the matrix-facing portion of the beta subunit, making it an accessible site for the rapid regulation of the ATP synthase complex assembly and phosphorylation-dependent modulation of its activity [68,70,71]. A decrease in the activity of the human ATP synthase has also been observed upon the phosphorylation of the gamma subunit (ATP5C1) by protein kinase C (PKC) [72]. Similar findings have been made in bovine heart mitochondria [73]. Interestingly, our phosphoproteomic data revealed a decreased level of Thr-85 phosphorylation in the gamma subunit of the ATP synthase in the AMPKα-overexpressing cells (Table 3). Despite the lack of data on the structure and phosphorylation-dependent regulation of ATP synthase activity in *Dictyostelium* mitochondria, it is tempting to speculate that the downregulation of phosphorylation of the specific sites of beta and gamma subunits in the HPF444 ATP synthase leads to its activation. This could maintain ATP synthase activity at the basal level, despite the significantly decreased abundance of the ATP synthase complex in the mutant mitochondria revealed by the 2-D PAGE proteomics in which beta, gamma and OSCP subunits were detected at greatly reduced levels (at fold changes −2.67, −1.47, −3.37, respectively) (Figure 4, Supplemental Table S4).

The phosphorylation of proteins imported from the cytosol into mitochondria can occur either before or during their import or inside mitochondria. In mammals, there are more than 30, while in yeast, at least 25 protein kinases are reported to be localized or functionally associated with mitochondria [62,63,74]. Here, we showed the presence of four different kinases associated with *Dictyostelium* mitochondria—GSK3, SvkA, SHKA, and SHKD (Table 2). In humans, the phosphorylation of the GSK-3B kinase at the Tyr-216 leads to the activation of this enzyme, and GSK-3B has been shown to translocate into mitochondria upon stress induction, where it is involved in the regulation of mitochondrial biogenesis and bioenergetics, mitochondrial motility, as well as mitochondria-induced apoptosis [75]. Here, the identification of the crucial phosphorylation site (Tyr-214) for the *Dictyostelium* GSK3 activity [44] suggests a similar activation of GSK3 through tyrosine phosphorylation under our conditions. However, this appears unrelated to AMPK, as we did not find any differences in the phosphorylation level between the AMPKα-overexpressing cells and the wild-type cells (Table 1 and Table 3).

The identification of SHKA, the orthologue of the human TAK1 kinase involved in the mitogen-activated protein kinase (MAPK) cascade pathway, in three independent *Dictyostelium* mitochondrial isolations, is of great importance. Even though there is no experimental data on the presence of TAK1 in mitochondria, there is a possibility of the transient mitochondrial colocalization of this kinase upon specific conditions. Recently, Hindi et al. [76] have shown that the deletion of TAK1 leads to the accumulation of enlarged or greatly interconnected mitochondria in the skeletal muscles of adult mice through the activation of AMPK. Furthermore, several studies revealed the direct role of TAK1 in the phosphorylation of AMPK [77,78] and the signalling of AMPK to activate TAK1 [79,80]. Our phosphoproteomic analysis revealed, in the HPF444 mitochondrial fractions, a lowered phosphorylation level of the Tyr-525 residue of SHKA, which corresponds to Tyr-533 of its human orthologue (Table 1, Table 3 and Table S2).

Previous studies have shown that the overexpression of the *D. discoideum* catalytic AMPKα subunit led to a decrease in the growth rate, severe morphological and developmental alterations, as well as increased intracellular levels of ATP and enhanced mitochondrial biogenesis [11,27]. A link between the AMPK activity and the maintenance of mitochondrial integrity/morphology and mitochondrial biogenesis has been well recognized [16]. However, a mechanism underlying AMPK-mediated mitochondrial biogenesis is not well understood. Activated AMPK phosphorylates and activates key protein substrates of catabolic pathways and inactivates proteins involved in biosynthetic pathways to restore cellular energy balance. There are now more than 60 validated downstream AMPK protein targets, including mitochondrial proteins (MFF, ACC2, cytochrome c, A-kinase anchor protein 1 (AKAP1)) [19,20,21,81,82,83]. The recognition of which *Dictyostelium* mitochondrial proteins are potential downstream targets that respond to the increased activity of AMPK appears to be challenging due to the identification of a large number of mitochondrial phosphoproteins of unknown function. Assuming that AMPK directly or indirectly phosphorylates a mitochondrial or mitochondria-related protein target to trigger mitochondria into biogenesis, we searched for proteins characterized by an increased phosphorylation level. We found only four such phosphoproteins (LETM1, S5 DRBM domain-containing protein, and two uncharacterized proteins), which could be potential substrates for AMPK activity (Table 3). By similarity to other organisms, LETM1 is presumably located in the *Dictyostelium* inner mitochondrial membrane, while S5 DRBM domain-containing protein resides in the matrix. This localization would hinder direct recognition by AMPK. However, the identification of enhanced phosphorylation of the LETM1 Thr-282 in the HPF444 mitochondria and the discovery that, in humans, LETM1 regulates mitochondrial calcium transport via PINK1-dependent phosphorylation [32] make this protein a potential regulatory target in the process of mitochondrial biogenesis. The role of mitochondrial calcium uptake in promoting mitochondrial biogenesis and mitochondrial network dynamics has recently been described [84,85].

In unicellular eukaryotes, including slime moulds, such as *D. discoideum*, cell growth is regulated mainly by the availability of nutrients, unlike metazoan organisms, which additionally require growth factors and cytokines to regulate nutrient supply and its availability for cell growth and proliferation [86]. In our studies, the wild-type and AMPKα-overexpressing cells were cultivated in a nutrient-rich medium, yet the decreased growth rate was observed with chronically active AMPK, which can mimic a situation in which there is a lack of nutrients. There are two major and highly conserved nutrient-sensing signalling pathways controlling cellular growth. One is AMPK itself and the second is the mTORC1 (mechanistic target of rapamycin complex 1) pathway. While AMPK suppresses cell growth by activating catabolic processes and inhibiting anabolic processes, mTORC1 promotes cell proliferation and growth by enhancing protein translation, lipid and nucleotide synthesis, and blocking autophagy [87]. In most eukaryotes, including *Dictyostelium*, there are two structurally and functionally distinct multiprotein mTOR complexes, rapamycin-sensitive mTORC1 and rapamycin-insensitive mTOR complex 2 (mTORC2), and both with mTOR serine/threonine protein kinase as a central catalytic component of the signalling hub [88,89,90].

There is an increasing body of evidence of the intrinsic crosstalk between the mTORC1 and AMPK pathways. The AMPK activation inhibits mTORC1, and this would have a major effect on conserving energy [87]. For example, mammalian AMPK directly phosphorylates two serines (Ser-722 and Ser-792) of RAPTOR, a structural component of the mTORC1, and these modifications inhibit mTORC1 activity [91]. One of the significant players of the mTORC1 pathways is LAMTOR1, a protein anchored to the lysosomal membrane and a crucial component of the Ragulator complex, which acts as a scaffold for the amino acid-activated mTORC1 [92,93]. It has been recognized that there is a pool of AMPK that permanently resides on the lysosomal membrane [94]. Upon glucose starvation, lysosome-bound AMPK is activated via the AXIN/LKB1 protein complex, and further, the AXIN/LKB1-AMPK complex binds to LAMTOR1, leading to the dissociation and inhibition of mTORC1. In our work, multiple phosphorylation sites of LAMTOR1 have been detected, and among them, three showed lowered phosphorylation levels in the mutant cells (Table 1 and Table 3). It is currently unknown whether the phosphorylation/dephosphorylation of LAMTOR1 affects the stability and activity of the mTORC1 complex.

Nutrients and, specifically, amino acids are the most effective stimuli for mTORC1 activation. In mammals, the Golgi-localized GTPase Rab1A acts as a conserved regulator of serine and glutamine signalling, which stimulates its binding to mTORC1 and the activation of the complex on the Golgi apparatus [95]. Interestingly, for its normal function, Rab1A needs to be phosphorylated. Levin et al. [96] demonstrated that the phosphorylation of Rab1 is executed by TAK1 kinase at the Thr-75 residue, which is essential for Rab1 activation. Our phosphoproteomic studies revealed a decreased level of phosphorylation of Rab1A at Ser-76, indicating that this protein has a reduced function in the HPF444 mutant cells (Table 3).

**Figure 6:**
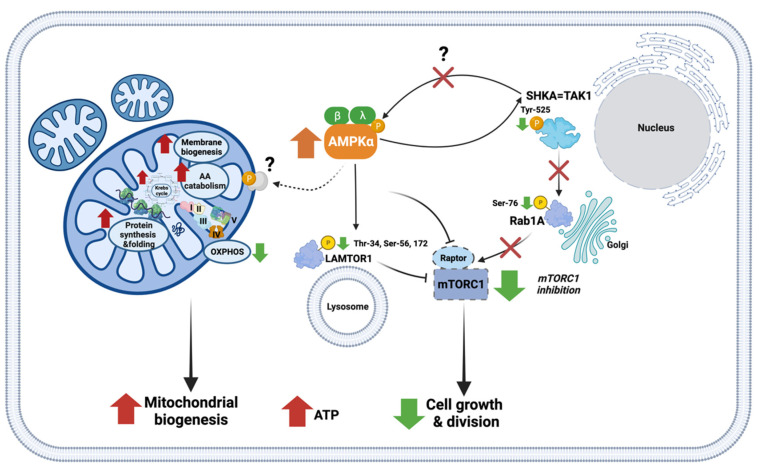
A hypothetical scenario of the events leading to enhanced mitochondrial biogenesis and reduced growth rate of *D. discoideum* vegetative cells in the presence of chronically active catalytic AMPKα subunit. The directions of short green, red and orange arrows specify either change in the protein abundance/phosphorylation level or direction of a specific process in the presence of chronically active AMPKα subunit. Black lines ending with arrowheads indicate activating effects, while perpendicular lines indicate inhibiting effects. Black dotted lines indicate hypothetical processes. Chronically active AMPK leads to the inhibition of the mTORC1 pathway via dephosphorylation-dependent events including the SHKA-Rab1A-mTORC1 route and/or the AMPK-LAMTOR1-mTORC1 pathway, which in consequence results in a decrease in cell growth and division. Enhanced mitochondrial biogenesis is induced by not yet identified mitochondrial protein target which upon direct or indirect phosphorylation by AMPK leads to dramatic changes in the abundance and/or phosphorylation-dependent activity of mitochondrial proteins involved in energy metabolism, protein synthesis and membrane biogenesis. This in turn causes the presence of increased mitochondrial mass and level of cellular ATP. SHKA, dual-specificity protein kinase SHKA; Ras1A, Ras-related protein Ras1A; mTORC1, mechanistic target of rapamycin complex 1; LAMTOR1, Ragulator complex protein Lamtor1. Created with BioRender.com.

In this study, we found differentially phosphorylated proteins involved in the AMPK/mTORC1 signalling pathways (LAMTOR1, Rab1A, SHKA) in the *Dictyostelium* mitochondrial fractions and observed changes in the abundance of mitochondrial proteins involved in energy metabolism, protein synthesis, and membrane biogenesis. This suggests a possible mechanism involving an interplay between the AMPK and mTORC1 pathways in the AMPKα-overexpressing *Dictyostelium* cells, resulting in reduced cellular growth rate accompanied by enhanced mitochondrial biogenesis. The crosstalk between AMPK and mTORC1 in *Dictyostelium* during vegetative growth and development has been recently described [97]. In healthy cells, the activities of mTORC1 and AMPK are coordinated in a network of regulatory pathways. Here, we propose that chronically active AMPK leads to the inhibition of the mTORC1 pathway via dephosphorylation-dependent events including the SHKA-Rab1A-mTORC1 route and/or the AMPK-LAMTOR1-mTORC1 pathway (Figure 6). This could combine with AMPK’s direct inhibitory phosphorylation of the RAPTOR subunit of mTORC1 to inhibit its activity and produce a decrease in cell growth and division [97]. mTORC1 is known for stimulation of expression of OXPHOS proteins [98,99]; hence, it is likely that the downregulation of OXPHOS components in AMPKα-overexpressing *Dictyostelium* cells results from the inhibition of mTORC1 activity. Enhanced mitochondrial biogenesis is induced by as yet unidentified mitochondrial protein targets, which, upon direct or indirect phosphorylation by AMPK, leads to dramatic changes in the abundance and/or phosphorylation-dependent activity of mitochondrial proteins involved in energy metabolism, protein synthesis, and membrane biogenesis. These changes, in turn, lead to an increase in mitochondrial mass/membrane potential and cellular ATP levels [11], as well as an increase in mitochondrial basal and maximum respiration rates (Figure 5) in the AMPKα-overexpressing cells.

## 4. Materials and Methods

### 4.1. D. discoideum Strains and Culture Growth Conditions

The experiments were conducted with the *D. discoideum* wild-type (WT) strain, A × 2, and the AMPKαT transformant overexpressing the catalytic domain of the AMPKα subunit (*snfA*) derived therefrom (strain ID: DBS0238735, www.dictybase.org). The studied AMPKαT strain was previously described in Bokko et al. [11]. In this work, the stable clone HPF444 overexpressing AMPKαT has been used.

The *D. discoideum* cells were axenically grown in HL-5 medium (Formedium, Hunstanton, Norfolk, UK) [100], supplemented with 100 μg/mL ampicillin, and for the transformed line, additionally with 20 μg/mL geneticin (G-418). Cell suspensions were cultured at 22 °C on a rotary shaker at 150 rpm. The generation time of continuously agitated wild-type and HPF444 cultures was approximately 8–9 h and 10–11 h, respectively.

### 4.2. Mitochondria Isolation

Mitochondria were isolated from vegetative cells of WT and HPF444 collected at densities of 1.5–2.0 × 10^6^/mL or 1.8–2.2 × 10^7^/mL (exponential and stationary phase of growth, respectively). The cells were harvested by centrifugation at 700× *g* for 5 min at 4 °C and washed twice in cold Sorensen phosphate buffer (15 mM KH_2_PO_4_, 2 mM Na_2_HPO_4_, ×7H_2_O, pH 6.0). Cells were resuspended in ice-cold STEB buffer (200 mM sucrose, 20 mM Tris-Cl, 1 mM (ethylene glycol-bis(β-aminoethyl ether)-N,N,N′,N′-tetraacetic acid), EGTA, pH 8.0, supplemented with 0.2% defatted bovine serum albumin, BSA) in the amount of 1 mL STEB/1 × 10^8^ cells and lysed in a waring blender. The extract of cells was centrifuged for 20 min at 27,000× *g* and 4 °C. The obtained pellet was then resuspended in ice-cold STEB buffer with BSA and centrifuged for 4 min at 400× *g* and 4 °C. The resulting supernatant was centrifuged for 10 min at 16,000× *g* and 4 °C. The obtained mitochondrial pellet was washed twice in a STEB buffer without BSA by centrifugation at 16,000× *g* for 10 min and 4 °C. Isolated crude mitochondria were resuspended at a density of 10 mg of proteins per 1 mL of STEB without BSA, applied onto a three-step sucrose gradient (4 mL 23%, 4 mL 32%, 2.5 mL 60%), and centrifuged at 150,000× *g* for 1 h at 4 °C. Purified mitochondria were washed twice in STEB at 16,000× *g* for 10 min and 4 °C to remove excess sucrose. The protein concentration of the mitochondrial samples was evaluated with the DC Protein Assay (Bio-Rad, Richmond, CA, USA), based on Lowry’s method [101].

### 4.3. Protein Sample Preparation for Phosphoproteome Identification with Mass Spectrometry

The mitochondrial pellets of WT and HPF444 that have been obtained from three independent biological replicates of the cells from the exponential phase of growth were resuspended in lysis buffer (8 M urea in 50 mM triethylammonium bicarbonate, pH 8.5, 1 mM sodium orthovanadate, one tablet of Complete mini EDTA-free mixture (Roche Applied Science, Mannheim, Germany), one tablet of PhosSTOP phosphatase inhibitor mixture (Roche Applied Science, Mannheim, Germany) per 10 mL of lysis buffer and lysed by 10 rapid passages through a 23-gauge hypodermic syringe needle and by sonication on ice. After centrifugation at 20,000× *g* for 30 min at 4 °C, the protein concentration was determined using the Bradford assay (Pierce, Rockford, IL, USA). Proteins were reduced with 2 mM dithiothreitol (DTT) at 56 °C for 25 min and then alkylated with 4 mM iodoacetamide at room temperature for 30 min in the dark. Proteins were then reduced again with 2 mM DTT at room temperature to prevent over-alkylation. A first enzymatic digestion step was performed in 8 M urea lysis buffer using Lys-C at 37 °C for 4 h (enzyme/substrate ratio 1:50). The samples were then diluted four times with 50 mM triethylammonium bicarbonate, pH 8.5, and digested overnight at 37 °C using trypsin (enzyme/substrate ratio 1:50). The digestion was quenched with 5% formic acid. The resulting peptides were chemically labelled using stable isotope dimethyl labelling as described before [102]. The protein digests from the wild-type and HPF444 were labelled as “Light” (L) and “Heavy” (H), respectively. An aliquot of each label was measured on a regular LC-MS/MS run, and samples were mixed 1:1 (L:H) based on their peptide intensities and dried down.

### 4.4. Phosphopeptide Enrichment

The specific enrichment of phosphopeptides before mass spectrometry analysis was prepared using monodisperse microsphere-based immobilized titanium (IV) ion affinity chromatography (Ti_4_^+^-IMAC). Ti_4_^+^-IMAC material was prepared as described in Zhou et al. [103]. The prepared Ti_4_^+^-IMAC beads were loaded onto GELoader tips (Eppendorf, Hamburg, Germany) using a C8 plug to approximately 1–2 cm length of material. The enrichment procedure consisted of the following steps: first, the Ti_4_^+^-IMAC material was pre-equilibrated twice with 50 μL of Ti_4_^+^-IMAC loading buffer (80% acetonitrile (ACN), 6% trifluoroacetic acid (TFA)). During the next step, each SCX fraction was resuspended in 50 μL of loading buffer and loaded onto the equilibrated GELoader tips. Then, the Ti_4_^+^-IMAC material was washed with 50 μL wash buffer A (50% ACN, 0.5% TFA, 200 mM NaCl) and subsequently with 50 μL wash buffer B (50% ACN, 0.1% TFA). Bound peptides were first eluted by 30 μL of 10% ammonia into 30 μL of 10% FA. Finally, the remaining peptides were eluted with 2 μL of 80% CAN and 2% FA. The collected eluate was further acidified by adding 3 μL of 100% FA and stored at −80 °C for LC-MS/MS analysis.

### 4.5. 1-D SDS-PAGE and Pro-Q Diamond Staining for Phosphoprotein Detection in Polyacrylamide Gels

One hundred micrograms of mitochondrial proteins of WT and HPF444 isolated from the cells from the exponential and stationary phases of growth were solubilized in Laemmli sample buffer and resolved by 12% sodium dodecyl sulphate polyacrylamide gel electrophoresis (SDS-PAGE) [104]. Following electrophoresis, the gels were incubated overnight in a fixing solution (40% *v*/*v* methanol, 10% *v*/*v* acetic acid). After several washing steps in bidistilled water, the gels were then stained with 1× diluted Pro-Q Diamond phosphoprotein gel stain (Thermo Fisher Scientific, Waltham, MA, USA) for 3 h in the dark at room temperature to detect phosphorylated mitochondrial proteins. After staining, the gels were incubated in a Pro-Q Diamond destain solution (Thermo Fisher Scientific) for a total of 1.5 h (three times 30 min) in the dark. The stained gels were then visualized using the FLA 2000 scanner (Fuji Photo Film, Tokyo, Japan) at wavelengths of 532 nm and 580 nm for excitation and emission, respectively. Following imaging, the gels were further incubated in SYPRO Ruby protein gel stain (Thermo Fisher Scientific, Waltham, MA, USA) overnight in the dark at room temperature to detect total proteins. After staining, the gels were washed in a wash solution (40% *v*/*v* methanol, 7% *v*/*v* acetic acid) for 45 min in the dark. Imaging of total mitochondrial proteins was performed using the FLA 2000 scanner at wavelengths of 473 nm for excitation and 520 nm for emission.

### 4.6. Two-dimensional Polyacrylamide Gel Electrophoresis Analysis and Protein Identification

Mitochondrial proteins of WT and HPF444 isolated from three independent biological replicates of exponentially growing cells were extracted with a lysis buffer (8 M urea, 2 M thiourea, 2% *w*/*v* amidosulfobetaine-14, 20 mM DTT, 30 mM Tris-HCl, pH 8.5, protease inhibitor cocktail (Complete mini EDTA-free mixture, Roche Applied Science, Mannheim, Germany) and precipitated using the 2-D Clean-Up Kit (GE Healthcare, Uppsala, Sweden) as described previously [105]. Four hundred micrograms of mitochondrial protein samples were mixed with rehydration solution (7 M urea, 2 M thiourea, 2% *w*/*v* amidosulfobetaine-14, 2% *v*/*v* IPG buffer 4–7 (GE Healthcare), 1.2% *v*/*v* De-streak solution (GE Healthcare, Uppsala, Sweden), 1% DTT) to a final volume of 340 μL and applied onto 18 cm ImmobilineTM DryStrips, pH 4–7 (GE Healthcare, Uppsala, Sweden). The mitochondrial proteins were resolved isoelectrically using an IPGphor isoelectric focusing unit (Amersham Pharmacia Biotech, Freiburg, Germany). Rehydration of the strips and isoelectric focusing (IEF) was conducted at 0 V for 1 h (rehydration), 30 V for 165 Vh (step), 60 V for 480 Vh (step), 200 V for 200 Vh (step), 500 V for 500 Vh (step), 8 kV for 8 kVh (gradient), 8 kV for 36 kVh (step) and 30 V for 150 Vh (step) to reach 45 kVh (total) at a maximum setting of 50 µA per strip. After IEF, the IPG strips were equilibrated for 25 min in 6 M urea, 50 mM Tris (pH 8.8), 2% *w*/*v* SDS and 30% *v*/*v* glycerol, supplemented with 65 mM DTT and then for a further 15 min in the same buffer except that DTT was replaced with 135 mM iodoacetamide [105]. After the equilibration step, the IPG strips were applied on the top of 12.5% acrylamide and 0.1% *w*/*v* N,N′-methylene-bis-acrylamide gels (22 × 20 cm). Second-dimensional electrophoresis was performed at 180 V for 6–7 h at 15 °C in a Protean II xi unit (Bio-Rad, Richmond, CA, USA). After electrophoresis, the gels were incubated for 2 h in a fixing solution (40% *v*/*v* methanol, 10% *v*/*v* acetic acid) and then stained overnight in the colloidal Coomassie G-250 solution at room temperature [106]. To enhance contrast, the Coomassie-stained gels were washed several times in bidistilled water and then incubated for 1 h in a destaining solution (10% *v*/*v* methanol, 2% *v*/*v* orthophosphoric acid) and stored in 5% *v*/*v* acetic acid. Three biological replicates of each mitochondrial protein sample were run independently on 2-D PAGE gels. The gels were scanned, and the generated TIFF images were analyzed using Delta 2-D Software 4.2 (DECODON, Greifswald, Germany) for normalization and statistical analysis. Protein spots that showed a significant difference in abundance between HPF444 vs. WT (fold difference of ±1.2, *p* ≤ 0.05.) were manually picked from the gels and designed for tryptic digestion and peptide extraction. The gel pieces were washed three times in a digestion buffer (50 mM triethylammonium bicarbonate, pH 8.5, 5% *v*/*v* ACN) and then incubated in 100% ACN to dehydrate the gels. In-gel tryptic digestion was performed with freshly activated trypsin (self-digestion-protected, sequencing-grade, Sigma-Aldrich, St.Louis, MO, USA) at a 12.5 µg/µL concentration in a digestion buffer. After rehydration of the gel pieces at 8 °C for 30 min, tryptic digestion was carried out overnight at 37 °C. The resulting peptides were extracted with 1% TFA for 30 min at room temperature with occasional shaking. A volume of 3 µL of the protein digest was transferred onto Prespotted AnchorChip plates with CHCA as a matrix, and the spots were briefly desalted with 10 mM dihydrogen ammonium phosphate in 0.1% TFA. The samples were analyzed by the Ultraflex II MALDI-TOF-TOF mass spectrometer (Bruker Daltonics, Bremen, Germany) using the TOF/MS-LIFT-MS/MS combined mode as described in Czarna et al. [105].

### 4.7. Seahorse Respirometry

A Seahorse Analyser (Agilent Technologies, Santa Clara, CA, USA) was used for the mitochondrial respiration assays as described previously [60]. *Dictyostelium* cells grown in HL-5 medium with shaking overnight were washed and resuspended in SIH (Formedium, Hunstanton, Norfolk, UK) media supplemented with 20 mM pyruvate and 5 mM glutamate, pH 7.4. Cells were then inoculated onto a 24-well cell culture plate, pre-treated with Matrigel, to ensure cell attachment during the assay. Basal oxygen consumption rates (OCR) were measured in the absence and presence of 10 μM N,N′-dicyclohexylcarbodimide (DCCD), ATP synthase inhibitor to estimate the contribution of the ATP-linked OCR (DCCD-sensitive OCR) and non-ATP-linked OCR (DCCD-resistant proton leak). Subsequently, 10 μM carbonyl cyanide 3-chlorophenol hydrazone (CCCP) was added to determine the maximum OCR. The mitochondrial inhibitors and reagents were then injected to estimate the contribution to the maximum OCR of each of the mitochondrial complexes, including alternative oxidase (AOX), in the following order: 20 μM rotenone, complex I inhibitor and either 10 μM antimycin A, complex III inhibitor or 1.5 mM benzohydroxamic acid (BHAM), AOX inhibitor. The residual (non-mitochondrial) respiration observed after complete blockage by mitochondrial electron transport inhibitors was included in the calculation of the respective OCR.

## Figures and Tables

**Table 1:** Phosphorylated mitochondrial and mitochondria-associated proteins identified in mitochondrial preparations from *D. discoideum* wild-type (WT) and AMPKα-overexpressing (HPF444) strain. UniProt, Universal Protein Resource database, protein ID; DictyBase, *Dictyostelium discoideum* genome database, protein ID; Protein, protein name (in brackets, *gene name*); Phosphopeptide(s), identified phosphorylated peptide sequence; “marked in red colour”, phosphorylated amino acid residue within the identified peptide; Phosphorylation site, phosphorylated amino acid in the protein sequence (Ser, serine; Thr, threonine; Tyr, tyrosine).

UniProt	DictyBase	Protein	Phosphopeptide(s)	Phosphorylation Site
**OXPHOS**				
Q9U3X4	DDB0214886	Succinate dehydrogenase (*sdhA)*	gEGGYLLNSsGER	Ser-300
Q54D07	DDB0238603	Cytochrome *c*1, heme protein (*cyc1)*	amAADtTVmDGPDSEGDmFER amAADTTVmDGPDsEGDmFER	Thr-104 Ser-112
Q1ZXP3	DDB0233077	Cytochrome *b-c*1 complex, subunit 6 (*uqcrh)*	gPIQEGcAsGcEk vkGPIQEGcAsGcEk	Ser-18
Q54V76	DDB0267111	Cytochrome *b-c*1 complex, subunit 8 (*uqcrq*)	qITYSVsPFQQk	Ser-17
Q54NW9	DDB0238608	Cytochrome *b-c*1 complex, subunit Rieske (*ucr)*	fITsDkIIVGDE	Ser-206
P30815	DDB0214995	Cytochrome *c* oxidase, subunit 4 (*cxdA)*	vGsPEFDk	Ser-55
P29505	DDB0191104	Cytochrome *c* oxidase, subunit 5 (*cxeA)*	hISsEGEVmYY	Ser-113
A9CLV8	DDB0350620	ATP synthase, subunit 4 *(atp4)*	dLsSDEITNSSTk dLSsDEITNSSTk dLSSDEItNSSTk	Ser-108 Ser-109 Thr-113
Q54DF1	DDB0237782	ATP synthase, subunit gamma *(atp5C1)*	vLGVVETADAFNTAtEPIEDR	Thr-85
Q54RA8	DDB0266798	ATP synthase, subunit O (OSCP) *(atp5O)*	tALEGDIDNsEk yLtLDESkN	Ser-133 Thr-290
Q55CS9	DDB0233951	ATP synthase, subunit beta *(atp5B)*	sLLDkESNEESTEVDYSk sLLDkEsNEESTEVDYSk sLLDkESNEEsTEVDYSk sLLDkESNEEStEVDYSk vIEDLNNPsLk aPPPFADLAPSAsILETGIk	Ser-58 Ser-64 Ser-68 Thr-69 Ser-128 Ser-231
**OXPHOS complex regulation and assembly**				
Q54ID0	DDB0305161	Cytochrome *c* oxidase copper chaperone *(cox17)*	sIAETNTTTEVAAPk	Ser-2
Q9GSE7	DDB0216175	F1F0-ATPase putative regulatory protein *(if1)*	kAGSQPTPNASSSANNsk	Ser-81
**Genome repair & maintenance**				
Q8MYF0	DDB0304673	Mitochondrial genome maintenance protein *(mgm101)*	iTEQQDDsEDIDIDDVVPPQLk	Ser-315
**Protein synthesis, folding & stabilization**				
Q54WN8	DDB0304956	Uncharacterized protein; probable mitochondrial small ribosomal subunit (*DDB_G0279527)*	eIIDQNPNDStDmPITR	Thr-846
Q54CA5	DDB0306787	S5 DRBM domain-containing protein, mitochondrial small ribosomal subunit	tIFEDGELDTPsVNSIR tIFEDGELDTPSVNsIR lGTVEHEELtFDHEk rFDEDYAEEsEILSQFSk eLsEYEEVIHQR	Ser-802 Ser-805 Thr-915 Ser-1006 Ser-1587
Q55GH1	DDB0306542	Protein similar to yeast tRNA threonylcarbamoyladenosine dehydratase 2 *(DDB_G0268496)*	sLNGGGGGGDDDGDNNNSsPSNQHIDk	Ser-51
Q54F93	DDB0232199	Mitochondrial-processing peptidase subunit alpha-2 (*mppA)*	vTFGNDESSTSIsNETAQYIGGESLk vTFGNDESSTSISNETAQYIGGESLkYSSGNsk	Ser-235 Ser-254
Q8I0H7	DDB0215366	Heat shock 70 kDa protein, mitochondrial (*mhsp70)*	dNTTEAEFtEkk	Thr-655
Q8MPA5	DDB0232124	Heat shock protein, Hsp20 domain-containing protein (*hspG7)*	sSTsPSSSTLDSk	Ser-96
C7G004	DDB0304476	Heat shock protein, DnaJ family protein *(DDB_G0304475)*	yIDNLIIPSSSSsDSGSGSGGSk yIDNLIIPSSSSSDsGSGSGGSk yIDNLIIPSSSSSDSGSGSGGsk	Ser-213 Ser-215 Ser-222
Q54Q31	DDB0232062	Prohibitin-2 (*phbB)*	sIsSLTGSk	Ser-91
**Transport**				
Q01501	DDB0185213	Mitochondrial outer membrane protein porin *(porA)*	yGsIVAVTDIk qILLSTLYTATsk	Ser-47 Ser-191
O97470	DDB0201558	Mitochondrial substrate carrier family protein ANT; ADP/ATP carrier protein *(ancA)*	dsLIGGTAGGVSk lLLQVQsASTQIAADk lLLQVQSAsTQIAADk lLLQVQSAStQIAADk lAADVGtGSAR lAADVGTGsAR lmGFEGGVGsE	Ser-16 Ser-44 Ser-46 Thr-47 Thr-150 Ser-152 Ser-308
Q54BF6	DDB0233888	Mitochondrial substrate carrier family protein N *(mcfN)*	aGDLtPSLFLk	Thr-6
Q54H87	DDB0346938	Uncharacterized protein (similar to *S. cerevisiae* mitochondrial phosphate transport protein PHO88m) (*DDB_G0289621*)	vTEINEsESSSEESEkEEk	Ser-216
Q86AV5	DDB0234131	Mitochondrial substrate carrier family protein X *(mcfX)*	gLSsNLIGIIPEk	Ser-87
Q54Y17	DDB0306880	LETM1 and EF-hand domain-containing protein (*DDB_G0278471)*	sGQtVTSDEVLk	Thr-282
**Metabolism**				
Q55GD7	DDB0306487	CDGSH iron-sulfur domain-containing protein (ortholog of human CISD3), similar to mitoNEET-related protein 2 (*DDB_G0267712)*	yNEETGLNDsPLk yNEETGLNDsPLkVEk kYNEETGLNDsPLk	Ser-75
Q555A3	DDB0203193	Carbonic anhydrase (*DDB_G0274643*)	lkENISLSTsN lkENISLStSN	Ser-273 Thr-272
Q55BA8	DDB0215348	Probable calnexin (*cnxA)*	eSVSIQDkPTIEsEESDESDEDNETTkk eSVSIQDkPTIESEEsDESDEDNETTk eSVSIQDkPTIESEESDEsDEDNETTk	Ser-510 Ser-513 Ser-516
**Signalling**				
P51136	DDB0185150	Glycogen synthase kinase-3 *(gskA)*	gETNVsYIcSR gETNVSyIcSR iLIkGETNVSyIcSR	Ser-213 Tyr-214
O61122	DDB0191176	Severin kinase *(svkA)*	sLsNSSQTTPVk	Ser-375
Q54RB7	DDB0191149	Dual specificity protein kinase SHKA *(shkA)*	aQLSGyIN	Tyr-525
Q54U31	DDB0230122	Dual specificity protein kinase SHKD *(shkD)*	fTQETFNPyDPYTN	Tyr-739
O00910	DDB0215388	Signal transducer and activator of transcription A *(dstA)*	rTAPVPVGGyEPLNS	Tyr-702
Q1ZXA8	DDB0231625	Protein similar to human Ragulator complex protein LAMTOR1 *(DDB_G0292160)*	nQASSSQQPSSSQtPSk nQASSSQQPSSSQTPsk dSEEQPQEVSYsQmR ecGELVVFFGNsLk	Thr-34 Ser-36 Ser-56 Ser-172
P34139	DDB0191476	Ras-related protein Rab1A *(rab1A)*	tITSsYYR	Ser-76

**Table 2:** Kinases identified in mitochondrial preparations from *D. discoideum* WT and HPF444 strains. UniProt, Universal Protein Resource database; DictyBase, *Dictyostelium discoideum* genome database; Protein, protein name; Mitochondrial preparations, number of mitochondrial preparations in which a given kinase and its phosphorylation site were identified.

UniProt	DictyBase	Protein	Kinase Group	Mitochondrial Preparations
P51136	DDB0185150	Glycogen synthase kinase-3	CMGC kinases	2
O61122	DDB0191176	Severin kinase	STE kinases	2
Q54RB7	DDB0191149	Dual specificity protein kinase SHKA	TKL kinases	3
Q54U31	DDB0230122	Dual specificity protein kinase SHKD	TKL kinases	1

**Table 3:** Differentially phosphorylated proteins identified in mitochondrial preparations from the *D. discoideum* HPF444 strain in comparison to WT in at least two biological replicates or two peptides. P-site, phosphorylation site (number of phosphorylated amino acid residue within the identified peptide). Detailed quantitative analysis is presented in Table S3.

DictyBase	Protein	P-Site
**Proteins with up-regulated phosphorylation**		
DDB0306880	LETM1 and EF-hand domain-containing protein	Thr-282
DDB0306787	S5 DRBM domain-containing protein	Ser-1587
DDB0306113	Uncharacterized protein	Thr-65
DDB0306695	Uncharacterized protein	Thr-367
**Proteins with down-regulated phosphorylation**		
DDB0238603	Cytochrome *c*1, heme protein	Ser-112
DDB0237782	ATP synthase, subunit gamma	Thr-85
DDB0233951	ATP synthase, subunit beta	Thr-69
DDB0215366	Heat shock 70 kDa protein, mitochondrial	Thr-655
DDB0304476	Heat shock protein, DnaJ family protein	Ser-213
DDB0201558	Mitochondrial substrate carrier family protein ANT	Ser-152, 308
DDB0234131	Mitochondrial substrate carrier family protein X	Ser-87
DDB0306487	Similar to mitoNEET-related protein 2	Ser-75
DDB0215348	Probable calnexin	Ser-513
DDB0191149	Dual specificity protein kinase SHKA	Tyr-525
DDB0231625	Protein similar to human Ragulator complex protein LAMTOR1	Thr-34, Ser-56, 172
DDB0191476	Ras-related protein Rab-1A	Ser-76
DDB0304899	Uncharacterized protein	Ser-290
DDB0347754	Uncharacterized protein	Ser-106, 107
DDB0305799	Uncharacterized protein	Ser-61, 536
DDB0302557	Uncharacterized protein	Thr-52
DDB0307109	Uncharacterized protein	Ser-647, 648, 650
DDB0306527	Uncharacterized protein	Ser-314
DDB0346952	Uncharacterized protein	Ser-230
DDB0232257	Uncharacterized protein	Ser-473
DDB0306348	Uncharacterized protein	Ser-128, Thr-130
DDB0346952	Uncharacterized protein	Ser-230
DDB0307677	Uncharacterized protein	Ser-301
DDB0306179	Uncharacterized protein	Ser-333
DDB0191444	Myosin-2 heavy chain	Ser-1636, 1637
DDB0191351	Myosin IB heavy chain	Tyr-334
DDB0185146	Myosin regulatory light chain	Ser-14
DDB0214946	Probable ATPase, P-type ATPase	Ser-22
DDB0232252	Crt homolog 3	Ser-438
DDB0191505	Vacuolin-A	Ser-14
DDB0234063	Phospholipid-translocating ATPase	Ser-86

## Data Availability

The data presented in this study are available on request from the corresponding author. The data are not yet publicly available due to the fact that the proteomics data were acquired and stored by a different organization and has not been deposited yet. The goal is having the data either deposited in a repository, or included as supplementary data, in case of publication.

## Supplementary Materials

The Supplementary Materials are available online at https://www.mdpi.com/article/10.3390/ijms222111675/s1.
