# Supplementary material for: Chronic Activation of AMPK Induces Mitochondrial Biogenesis through Differential Phosphorylation and Abundance of Mitochondrial Proteins in Dictyostelium discoideum
Source: Int J Mol Sci. 2021 Oct 28;22(21):11675. doi: 10.3390/ijms222111675 (PMC8584165; doi:10.3390/ijms222111675)
Supplement: Supplementary file 1 [file ijms-22-11675-s001.zip › Table S2.pdf]

Table S2. Phosphosites identified in mitochondrial and mitochondria-associated proteins from *D. discoideum* and corresponding amino acids of human orthologues matching the identified phosphosites.
UniProt, Universal Protein Resource database, protein ID; Protein, protein name (in brackets, *gene name*); Phosphorylation site, the phosphorylated amino acid in the *D. discoideum* protein sequence (Ser, serine; Thr, threonine; Tyr, tyrosine). *no AA*, no matching amino acid in the human orthologue was found after sequence alignment of two homologous proteins; * an asterisk indicates a position of Ser, Thr, or Tyr in the human orthologue, matching to the phosphorylated residue in *D. discoideum* protein.

D. discoideum			**Human orthologue**	
**UniProt**	**Protein**	**Phosphorylation site**	**UniProt**	**Corresponding AA**
OXPHOS				
Q9U3X4	Succinate dehydrogenase (*sdhA)*	Ser-300	P31040	Gln-322
Q54D07	Cytochrome *c*1, heme protein (*cyc1)*	Thr-104<br>Ser-112	P08574	Val-152<br>Glu-160
Q1ZXP3	Cytochrome *b*-*c*1 complex, subunit 6 (*uqcrh)*	Ser-18	P07919	Glu-41
Q54V76	Cytochrome *b*-*c*1 complex, subunit 8 (*uqcrq*)	Ser-17	O14949	Ser-20*
Q54NW9	Cytochrome *b*-*c*1 complex, subunit Rieske (*ucr)*	Ser-206	P47985	Asp-268
P30815	Cytochrome *c* oxidase, subunit 4 (*cxdA)*	Ser-55	P13073	*no AA*
P29505	Cytochrome *c* oxidase, subunit 5 (*cxeA)*	Ser-113	P10606	*no AA*
A9CLV8	ATP synthase, subunit 4 *(atp4)*	Ser-108<br>Ser-109<br>Thr-113	*unknown*	

Q54DF1	ATP synthase, subunit gamma<br>*(atp5C1)*	Thr-85	P36542	Ile-82
Q54RA8	ATP synthase, subunit O (OSCP)<br>*(atp5O)*	Ser-133<br>Thr-290	P48047	Ser-86*<br>*no AA*
Q55CS9	ATP synthase, subunit beta<br>*(atp5B)*	Ser-58<br>Ser-64<br>Ser-68<br>Thr-69<br>Ser-128<br>Ser-231	P06576	*no AA*<br>*no AA*<br>*no AA*<br>*no AA*<br>Glu-87<br>Glu-181
OXPHOS complex regulation and assembly				
Q54ID0	Cytochrome *c* oxidase copper chaperone<br>*(cox17)*	Ser-2	Q14061	Gly-3
Q9GSE7	F1F0-ATPase putative regulatory protein<br>*(if1)*	Ser-81	Q9UII2	*no AA*
Genome repair & maintenance				
Q8MYF0	Mitochondrial genome maintenance protein<br>*(mgm101)*	Ser-315	probable<br>Q9BQP7	Leu-213
Protein synthesis, folding & stabilization				
Q54WN8	Uncharacterized protein; probable mitochondrial small ribosomal subunit (*DDB_G0279527)*	Thr-846	*unknown*	
Q54CA5	S5 DRBM domain-containing protein, mitochondrial small ribosomal subunit	Ser-802<br>Ser-805<br>Thr-915<br>Ser-1006<br>Ser-1587	P15880	*no AA*<br>*no AA*<br>*no AA*<br>*no AA*<br>*no AA*
Q55GH1	Protein similar to yeast tRNA threonylcarbamoyladenosine dehydratase 2<br>*(DDB_G0268496)*	Ser-51	*unknown*	

Q54F93	Mitochondrial-processing peptidase subunit alpha-2 (*mppA*)	Ser-235<br>Ser-254	Q10713	Asp-287<br>Thr-318*
Q8I0H7	Heat shock 70 kDa protein, mitochondrial (*mhsp70)*	Thr-655	P38646	*no AA*
Q8MPA5	Heat shock protein, Hsp20 domain-containing protein (*hspG7)*	Ser-96	*unknown*	
C7G004	Heat shock protein, DnaJ family protein *(DDB_G0304475)*	Ser-213<br>Ser-215<br>Ser-222	probable P31689	Gly-228<br>Gln-230<br>Gly-237
Q54Q31	Prohibitin-2 (*phbB)*	Ser-91	Q99623	Ser-91*
Transport				
Q01501	Mitochondrial outer membrane protein porin *(porA)*	Ser-47<br>Ser-191	P21796	*no AA*<br>Lys-200
O97470	Mitochondrial substrate carrier family protein ANT; ADP/ATP carrier protein *(ancA)*	Ser-16<br>Ser-44<br>Ser-46<br>Thr-47<br>Thr-150<br>Ser-152<br>Ser-308	P12235	Phe-12<br>His-40<br>Ser-42*<br>Lys-43<br>Lys-147<br>Ala-149<br>*no AA*
Q54BF6	Mitochondrial substrate carrier family protein N *(mcfN)*	Thr-6	Q00325	*no AA*
Q54H87	Uncharacterized protein (similar to *S. cerevisiae* mitochondrial phosphate transport protein PHO88m) (*DDB_G0289621*)	Ser-216	*unknown*	
Q86AV5	Mitochondrial substrate carrier family protein X *(mcfX)*	Ser-87	Q9H936	Val-71
Q54Y17	LETM1 and EF-hand domain-containing protein (*DDB_G0278471)*	Thr-282	O95202	Arg-299

Metabolism				
Q55GD7	CDGSH iron-sulfur domain-containing protein (ortholog of human CISD3), similar to mitoNEET-related protein 2<br>(*DDB_G0267712)*	Ser-75	P0C7P0	Ser-83*
Q555A3	Carbonic anhydrase<br>(*DDB_G0274643*)	Ser-273<br>Thr-272	*unknown*	
Q55BA8	Probable calnexin<br>(*cnxA)*	Ser-510<br>Ser-513<br>Ser-516	P27824	Gln-565<br>Glu-568<br>Lys-571
Signalling				
P51136	Glycogen synthase kinase-3<br>*(gskA)*	Ser-213<br>Tyr-214	P49841	Ser-215*<br>Tyr-216*
O61122	Severin kinase<br>*(svkA)*	Ser-375	Q9Y6E0<br>O00506	*no AA*<br>*no AA*
Q54RB7	Dual specificity protein kinase SHKA<br>*(shkA)*	Tyr-525	O43318	Tyr-533*
Q54U31	Dual specificity protein kinase SHKD<br>*(shkD)*	Tyr-739	P04049	*no AA*
O00910	Signal transducer and activator of transcription A<br>*(dstA)*	Tyr-702	P42224	Asp-694
Q1ZXA8	Protein similar to human Ragulator complex protein LAMTOR1<br>*(DDB_G0292160)*	Thr-34<br>Ser-36<br>Ser-56<br>Ser-172	Q6IAA8	*no AA*<br>Thr-30*<br>Pro-44<br>*no AA*
P34139	Ras-related protein Rab1A<br>*(rab1A)*	Ser-76	P62820	Ser-79*
