# Supplementary material for: Chronic Activation of AMPK Induces Mitochondrial Biogenesis through Differential Phosphorylation and Abundance of Mitochondrial Proteins in Dictyostelium discoideum
Source: Int J Mol Sci. 2021 Oct 28;22(21):11675. doi: 10.3390/ijms222111675 (PMC8584165; doi:10.3390/ijms222111675)
Supplement: Supplementary file 1 [file ijms-22-11675-s001.zip › Table S4.pdf]

Table S4. Comparative 2-D PAGE analysis of the *D. discoideum* WT and HPF444 mitochondrial proteomes. Protein spots with abundances differing between HPF444 and WT were identified by MS. Fold change in abundance of HPF444 *vs* WT with a cutoff of 1.2 and a Student's *t*-test value of $p \leq 0.05$. Spot ID, number of a protein spot on the 2-D gel; UniProt, Universal Protein Resource database; DictyBase, *Dictyostelium discoideum* genome database. Mw - molecular weight in Da; pI, theoretical isoelectric point.

Spot ID	UniProt	Gene name	DictyBase	Protein name	Mw (Da)	pI	HPF444 *vs* WT	
				PDH complex and Krebs cycle			**Fold change**	***p*-value**
ID732	Q86HX0	*pdhB*	DDB0229442	Pyruvate dehydrogenase E1, beta subunit	39214	5.6	-1.62242	0.0176711
ID233	P36413	*pdhC*	DDB0215387	Dihydrolipoamide acetyltransferase E2	69171	9.3	2.38276	0.0001569
ID468	Q54EW8	*lpd*	DDB0216232	Dihydrolipoamide:NAD oxidoreductase E3	52104	7.1	2.4384	0.001155
ID249	Q54XS2	*aco2*	DDB0230168	Aconitase, mitochondrial	83889	8.9	2.59069	0.0014612
ID330	Q9U3X4	*sdhA*	DDB0214886	Succinate dehydrogenase, flavoprotein subunit	69157	6.5	-1.26778	0.0426641
ID553	Q869S7	*scsB*	DDB0231358	Succinate-CoA ligase *GDP*-forming, beta subunit	46034	7.6	1.93509	0.0003164
ID511	Q55AI5	*scsC*	DDB0231357	Succinate-CoA ligase *ADP*-forming, beta subunit	48661	8.9	1.79377	3.5E-06
				OXPHOS				
ID512	Q23883	*nad7*	DDB0201599	NADH dehydrogenase, subunit 7	46917	5.7	1.27598	0.0234931
ID929	Q54NW9	*ucr*	DDB0238608	Ubiquinol-cytochrome-*c* reductase subunit (Rieske iron-sulfur protein)	23470	9.2	-2.19809	0.0199153
ID329	Q55CS9	*atp5B*	DDB0233951	ATP synthase beta chain, mitochondrial	71029	5.5	-2.6712	0.0006655
ID798	Q54RA8	*atp5O*	DDB0266798	Mitochondrial F1 complex ATP synthase, O subunit	33109	7.6	-3.37229	0.0012667

ID866	Q54DF1	*atp5C1*	DDB0237782	ATP synthase gamma chain	33665	8.7	-1.47753	0.0010368
ID1408	Q55F42	*atp5D*	DDB0238330	ATP synthase F1 delta	18611	6.4	1.14896	0.0461375
				Mitochondrial biogenesis				
ID473	Q8MYF0	*mgm101*	DDB0304673	Mitochondrial genome maintenance protein	42824	5.4	1.43463	0.0274771
ID197	Q55BP9	*DDB_G0270412*	DDB0267127	Mitofilin (Mic60)	76013	4.9	1.84083	0.02441
				Protein synthesis, folding and stabilization				
ID641	Q54G88	*DDB_G0290311*	DDB0346730	Mitochondrial 54S ribosomal protein L17	43123	6	1.49151	0.0034798
ID236	Q55D72	*DDB_G0269762*	DDB0306589	30S ribosomal protein S18	96462	5.2	2.95173	0.0115325
ID252	Q54HG8	*DDB_G0289469*	DDB0306148	30S putative ribosomal protein S15 P	71039	5.5	1.57562	0.0326533
ID187	Q55E94	*gfm1*	DDB0234169	Mitochondrial translation elongation factor G	82335	6.2	3.24476	0.000127
ID594	Q54HB2	*tufM*	DDB0235257	Mitochondrial elongation factor Tu domain-containing protein	46111	7.9	1.86833	0.0005981
ID642	Q54LV3	*tsfm*	DDB0266366	Mitochondrial elongation factor Ts	39848	9.2	2.07939	0.0004314
ID353	Q54J97	*hspA*	DDB0219929	Mitochondrial chaperonin 60	60094	5.8	1.33626	0.0513731
ID298	Q8I0H7	*mhsp70*	DDB0215366	Mitochondrial Hsp70	71603	5.8	1.65270	0.00680
ID246	Q86L04	*trap1*	DDB0185036	TNF receptor associated protein 1 (heat shock protein Hsp90 family protein)	80187	6.3	3.35696	0.0002822
ID445	Q4W6B5	*mppB*	DDB0231799	Mitochondrial processing peptidase, beta subunit	52822	5.2	1.33163	0.03552
				Metabolism				

ID275	Q54KE6	*mccA*	DDB0230065	Methylcrotonyl-CoA carboxylase	77326	6.8	1.7721	0.0018676
ID508	Q54X04	*DDB_G0279287*	DDB0232198	Cysteine desulfurase, mitochondrial precursor	50164	7.8	1.62787	0.0016966
ID587	Q54W88	*ivdA*	DDB0230189	Isovaleryl-CoA dehydrogenase, mitochondrial	45383	7.6	1.54209	0.0037083
ID1241	Q55FN7	*bkdB*	DDB0230185	3-methyl-2-oxobutanoate dehydrogenase	41041	7.8	-3.13596	0.0011963
ID224	Q553S7	*pccA*	DDB0230063	Propionyl-CoA carboxylase, alpha subunit	80344	6.3	3.28394	0.0001971
ID358	Q8T131	*pccB*	DDB0235357	Propionyl-CoA carboxylase, beta subunit	61107	7	3.06013	0.0037501
ID584	Q54RR5	*acadsb*	DDB0237707	Acyl-CoA dehydrogenase, short/branched chain	45343	9.2	1.3649	0.0153942
ID589	Q55D79	*DDB_G0269756*	DDB0190529	3-hydroxyisobutyryl-CoA hydrolase	67251	6.1	-1.30973	0.0404062
ID625	Q54I98	*smt1*	DDB0237965	Putative delta-24-sterol methyltransferase	39819	6.2	-3.24497	0.0012024
ID267	Q551V0	*DDB_G0276305*	DDB0231480	Aldehyde dehydrogenase	69360	6.9	2.21013	0.0236746
ID491	P54872	*hgsA*	DDB0219924	Hydroxymethylglutaryl-CoA synthase	53992	9.1	2.60709	0.0017778
ID630	Q54Q74	*clybl*	DDB0302641	Citrate lyase subunit beta-like protein. Mitochondrial-like	40822	6.3	1.30856	0.0371834
				Miscellaneous				
ID645	Q55C71	*DDB_G0270752*	DDB0304715	Cytochrome b5 domain-containing protein	21415	6.3	1.24551	0.0294687
ID702	Q55EX9	*DDB_G0268948*	DDB0347390	Putative SAM dependent methyltransferase	33838	8.9	2.59167	0.0126562
ID163	Q9NKX1	*grp94*	DDB0215015	Glucose-regulated protein 94	87227	4.7	-2.37827	0.00732
ID282	Q54JL8	*DDB_G0287967*	DDB0252598	Hypothetical protein	12798	10.4	4.20793	0.000114

ID495	Q54BU5	*DDB_G0293420*	DDB0349095	Hypothetical protein	52710	6.3	2.11413	0.0159865
ID573	Q55EC2	Q55EC2	DDB0306113	Hypothetical protein	49771	6.1	-1.83914	0.0280075
